# New tools for a new age: An evolution or revolution in higher education?

**DOI:** 10.12688/f1000research.7533.1

**Published:** 2015-12-23

**Authors:** Roslyn Gleadow, Melissa Honeydew, Allie Ford, Bronwyn Isaac, Kirsti Abbott

**Affiliations:** 1 School of Biological Sciences, Monash University, Victoria, 3800, Australia; 2 School of Biomedical Science, Monash University, Victoria, 3800, Australia; 3 Faculty of Science, Monash University, Victoria, 3800, Australia; 4 Peninsula Library, Monash University, Victoria, 3800, Australia; 5 School of Environmental & Rural Science, University of New England, New South Wales, 2351, Australia

**Keywords:** higher education, science communication, communication, collaboration, social media

## Abstract

In this paper we describe how digital technologies can be used to enhance collaboration and student engagement in a large, multicampus undergraduate science unit. Four innovations developed and implemented over a period of eight years are described: use of electronic whiteboards, on-line discussion forums, social media and blogs. In showing the intermediate steps in the evolution of the use of digital and communication technologies, we demonstrate that to be effective, good educational principles are paramount.

## Introduction and rationale

In the past decade we have witnessed a revolution in the use of digital media. This is epitomised by an image that circulated various social media platforms, and originally posted on Instagram by NBC news comparing a photo from the inauguration of Pope Benedict in 2005 with one from the inauguration of Pope Francis in 2013. In the first, everyone is looking up and around, with only one small flip phone visible; in the second, there is a sea of iPads and smart phones lighting people’s faces and most people are looking at the Pope through a screen. The ability to easily share information with others across and space and time is unprecedented. The fact that the first photo may have been taken shortly after John Paul II’s death (also in 2005) rather than Pope Benedict’s inauguration also shows how easy it is for misleading information to “go viral” and impact the global perception of an event. This revolution in information access and sharing has huge implications for higher education when knowledge, not just perception, is at stake. It means not only that students can take seemingly credible courses from anywhere in the world through Massive Open Online Courses (MOOCs ) or enrolment in virtual classrooms, but that they also can have more control over the content they access and their schedules.

Learning primarily using digital technology tends to emphasise the acquisition of knowledge, and suits some students’ study styles. Unless this is understood, there may be reduced opportunities for social engagement and networking – factors that are an important aspect of student engagement, the sharing of ideas, and creating meaning for the learner ( Conrad & Donaldson, 2004). Social interactions typically happen during on-campus learning in traditional face-to-face lessons. Despite this, more and more students studying on campus prefer to listen online rather than attend lectures in person, to save travel time, schedule education around paid work, and fit in with other commitments ( Gleadow 2009, unpublished conference report), but these choices often reduce opportunities for collaboration.

We teach a course on scientific practice and communication. This unit is offered at a traditional research university where blended learning models are encouraged. We combine face-to-face teaching - using a mixture of lectures and tutorials - with online activities and value social engagement, networking and collaboration between students from different disciplines and backgrounds. The course is compulsory for all science students and runs throughout the academic year. Approximately 700 students are enrolled each semester, spread across the main campus in Melbourne, at a campus in Kuala Lumpur, Malaysia and, until recently at a regional campus, including a cohort of distance education students. An advanced form of the same unit is also delivered to a small group of 30–40 talented students each semester. The content of the unit deals with the nature and origins of science, ethical practice, science literacy, and various aspects of science communication.

Engagement of students studying at all levels is generally higher when they can work with their peers, work with technology, have control over their own learning and see that the teachers themselves are engaged ( Wolpert-Gawron, 2012; Zepke & Leach, 2010). We have endeavoured to improve student engagement and teamwork for those students choosing to learn primarily in the online environment using a combination of social media, collaborative document tools and, for in class use, both tablet computers and electronic whiteboards. This has evolved over a period of eight years ( Table 1). In this paper we outline 1) a chronology of when and how these digital technologies were introduced to students, 2) ways in which these technologies have changed, and are continuing to change, the way we teach and 3) their effectiveness, because not everything we trialled has been effective. In doing so, we show that the introduction of new teaching tools has been an evolution of earlier styles of learning, but compared to ‘then’ what we do ‘now’, looks like a revolution. The paper is based on the Keynote lecture given by one of us (RG) at the SEB Digital tools for teaching and communicating science, in London, December 2014, described by ( Scott, 2015).

**Table 1.** Timeline for introduction of technologies to enhance online interaction and collaboration in this case study.

2005	● PDFs of lecture slides, lecture summaries, workbook notes and links to reading are made available on line through LMS
	● Lectures recorded and released via Podcasting
2006	● Introduction of paperless assignment submission
2007	● Turnitin introduced to educate about plagiarism
	● Shift from email to online forums for all discussions, with certain areas quarantined for the different cohort
2008	● Trial of Wikis for collaborative project work by off campus students
2009	● Improvement of many administrative arrangements for students around group work and assignment submissions.
2009	● Signup sheets for assignment topics?
2010	● Students initiate small Facebook groups for private group work
	● Blogs initiated for advanced student cohort.
	● University moves student and staff accounts to gmail
2011	● Tablet PC trial
	● Podcasting updated to include images of slides
2012	● Facebook group for whole class initiated by students
	● Trial of live Twitter feeds in lectures ● Web based quizzes trialled in lecture
2013	● Electronic whiteboards trialled with advanced student cohort
2014	● Facebook page official
	● Trial of mind maps with advanced students
	● Electronic whiteboards placed in all tutorial rooms and used
2015	● Introduction of Mind maps
	● Introduction of on-line activities to replace lectures in some weeks
	● Restructure of LMS (see Gleadow *et al.*, 2015)

## Social media as a platform for instruction and student discussions

### Communicating on-line

Student work is centred on the learning management system (LMS), beginning with Blackboard in 2005, and moved to Moodle in 2010. LMSs, including ours, have traditionally functioned as a kind of information clearance house rather than educational space (see Gleadow *et al.*, 2015). There are discussion groups but these tend to be simple question and answer sites, where the teacher responds to specific questions. Such officially sanctioned forums are important, and their use can dramatically reduce the number of emails, but much of the discussion now takes place on social media, currently Facebook – there is one exception to this, the blogging that takes place in the advanced cohort (to be discussed later). Initially Facebook use was limited to students creating their own groups for working on joint assignments, but then in 2013, a proactive student created a group for the whole class and invited others to join via LMS. It was not until 2014, however, that students started approving membership of the group by lecturers and staff members, and since then Facebook has become an important medium for teaching and learning, further reducing the reliance on email. In the semester when we first used Facebook, 27 questions were posted on the LMS during the pre-examination study week and 148 questions were asked on Facebook. In the most recent semester there were over 300 posts over the whole semester on Facebook. Students answering each other’s questions is not only good for student learning ( Driver *et al.*, 1994), but also reduces the number of personal emails to the teachers.

**Figure 1.**
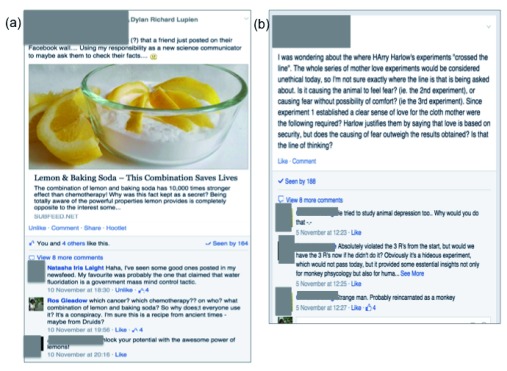
Examples screen grabs of the class Facebook page. ( **a**) Students here show engagement with the subject matter (pseudoscience) and extend beyond and apply what was presented in lectures. ( **b**) Discussion of examinable material from the lectures (on research ethics). Not only do students answer each other’s questions but go beyond, to express their own position on this ethical dilemma.

Students are mostly highly connected and comfortable on social media platforms. Informal surveys of students indicate that students consider Facebook “a given”. A show of hands in class indicated that over 90% of students are registered to use it. One attraction is that they can access it easily from a smart phone or other mobile device, and so the reach is much greater than with dedicated learning management systems – particularly when the LMS is not optimised for use on a variety of devices. This lowers the barriers to communication, and promotes informal language ( Figure 1). This switch to informal language has been a great help to our many international students who have been reluctant to post questions on the official forums because they report feeling embarrassed about making grammatical and spelling errors: over a third of students within the cohort do not speak English as their home language. Indeed, Yu *et al.* (2010) reported that students felt an enhanced social acceptance, and speedy adaptation to university culture through the use of social media in educational units.

Platforms such as the Facebook group give the students some control over the content and the way in which it is delivered. Educationally, this level of autonomy is a good thing, as it allows students to drive their own learning (Gleadow *et al.*, 1994). Students are also very good at answering each other’s questions using examples relevant to their cohort and/or sourcing relevant and entertaining videos that explain a point covered in lectures such as a video by Gentleman Thinker on critical thinking on the ‘Philosophy’ Youtube channel.

### Benefits of social media

Many students appreciate the almost instantaneous nature of receiving the answer to a query via Facebook because often one or two of the tutors are online too and it is simple for us to write a quick response. We are not advocating that teaching become a 24-hour day activity, but it certainly is convenient to deal with enquiries as they arise.

- *I really felt you went above and beyond in teaching the unit. Especially answering last minute frazzled assignment questions on Facebook. (Student feedback, 2015)*

### Disadvantages and problems in using social media

There are some drawbacks to using a public forum such as Facebook rather than relying on the functionality and accessibility built into the LMS. Not all students (or teachers) want to use mainstream social media platforms, nor should they be forced to. There may be privacy concerns, or philosophical opposition to them. Students may want their social spaces to remain separate from their online study resources, and make deliberate choices to disconnect from their studies when engaged in other activities. In our unit, we attempt to ensure that such students are not disadvantaged if they are not Facebook users by making all official announcements via LMS, and cross post on social media.

There is the danger, common to all social media platforms, that some students will use public forums inappropriately. It is important to bring the Facebook group under the umbrella of the University’s code of conduct. The administrator must set ground rules for the scope of the Facebook group in the group description, stating what topics are appropriate, defining what unacceptable behaviour is, and linking it to university policies regarding appropriate use of social media and IT. In our unit, these policies are discussed further as part of the ethics and communication components of workshops. Importantly, the course coordinator and the teaching team need to monitor the site, and have the power to remove posts that are not consistent with university policy. In the four semesters that this program has been operating we have only needed to remove posts by one student that could have been interpreted as derogatory. We have also observed a small number of cases of religious and political advocacy. Our strategies for dealing with these issues have so far been effective – the perpetrator is counselled via email and invited to a face-to-face meeting. At this point a simple reminder that they can be expelled from the course or even from the university for unacceptable behaviour is all that has been needed to ensure compliance. Thus, while using Facebook has numerous strengths, we need to learn from it, rather than rely on it. It is important to carefully consider the reasons students like and use specific platforms, so that we can incorporate these preferences into other things we do.

## Evolution from tablet PCs to electronic whiteboards

### Tablet PCs

In 2010 and 2011, we participated in a limited trial of tablet PCs run by the university ( Lindsay, 2012). Tablet PCs are normal laptop computers with screens that can be written on with a stylus, allowing annotation of existing documents, as well as creation of new ones. At this time, many students did not bring their personal computers to university, and collaborative software was not commonplace. The university was also developing its own collaborative software that allowed students (and their teachers) to work on the same document at the same time. Prior to this trial, any use of PCs required pre-booking of computer labs elsewhere on campus. The labs were available to the whole of our faculty, so it was often difficult to access them at the most appropriate times for specific course activities. When the tablet trial took place, we were able to access computers in our tutorial rooms every week for the first time. We made a deliberate choice to have one tablet PC per two students (rather than one each), to encourage collaboration.

The trial allowed us to make significant changes to our workshop structure and activities, for example students could search academic databases in class, rather than just watching a tutor demonstrate the process and then needing to try to remember it later. Shared brainstorming activities, collectively annotating journal articles and posters, and sharing appropriate items that each pair had found on the internet were new activities that added depth, and importantly 21 ^st^ century skills, to the existing curriculum. In one particular activity, working together to identify grammatical and stylistic errors in writing samples, students were enjoying themselves so much that they had to be asked to leave so the next class could begin; never before have we had to kick students out of a lesson on academic English.

Informal feedback from the students covered a number of themes. Many reported that the use of computers in their classes added more fun. Others liked that they could participate or look up information, as they needed it, rather than having to wait until they could get to the library. An important message came from some of the students from backgrounds other than English: they felt that they had a voice in the classroom. Some came from cultures where interrupting or contradicting someone else was rude. Many were concerned about their level of spoken English, or found that by the time they had worked out how to express their ideas in English, activities had moved on. Because of the change of pace brought about by the tablets, as students needed to research or develop ideas more, and the option to contribute in writing, these students reported feeling more confident, and more involved in their classes. This is consistent with the recommendations of Tanner (2013) for engaging students in learning. Gradually the tablet trial wound up, as other collaborative hardware and software began to appear on the market, so we took the lessons (and activities) from the small screen to larger screens.

### Electronic whiteboards

**Figure 2.**
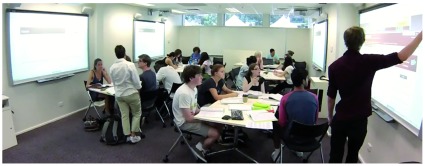
Tutorial rooms showing arrangement of electronic white boards in the tutorial rooms and layout of the furniture. Here students are accessing on-line databases to search for primary literature. There are approximately 600 students enrolled at this campus, with students broken into tutorial groups of 20–25. There are two rooms side-by-side to accommodate the large number of students.

The use of electronic interactive whiteboards has revolutionised the way that we educate students within the workshop setting. These screens, incorporating both electronic displays and traditional whiteboard capabilities allow for a more blended mode of teaching. Used appropriately, they can foster collaborative exchanges of ideas in small group work, and allows the students to become the teachers. Typically up to six students share a table and whiteboard, and we have four electronic interactive whiteboards in each tutorial room ( Figure 2). This setting creates an environment where anecdotally it seems to be easier for students to alternate between whole class activities with up to 25 students, and discussions with their smaller groups. It is often in these small groups where students are more confident to voice opinions and work effectively to develop content together.

Electronic whiteboards are highly versatile for use in a dynamic student-driven classroom environment. Collaborative small-group tasks range from interactive brainstorming of a concept relevant to lecture material, through to proof reading and editing a scientific poster before presenting it to the class, and even to the collaborative preparation of written documents, such as a cover letter addressing key selection criteria for a real science job. If necessary, the work can then be saved for future reference and emailed directly to group members from the classroom. Where students are given an open-ended group task, they use the technology available to them in a variety of ways that suit their learning style and expertise. For example, a small cohort of advanced students can quickly and collaboratively create visuals to communicate ideas for impromptu talks. They are able to conduct research and access materials from a variety of online sources and used the whiteboards in a variety of other ways as a platform to showcase the results of their work to the rest of the class. Each student brings with them knowledge of different tools, so that together their power to create and innovate is far greater than them working alone.

- *The new Interactive whiteboards [we used in] the unit aid learning and add stimulation for visual learners.* (Student feedback, 2014)

Access to a range of online resources, including the LMS and other suitable websites, is also easy from the electronic whiteboards. We encourage students to work together to develop skills in searching online databases, as well as identifying and effectively reading primary articles. Because the members of each group share a screen, rather than working on their own individual devices, there is increased transparency, collaboration and discussion. Teaching staff are also better able to observe *how* the group approaches each activity, monitor progress, and offer timely guidance and feedback, rather than needing to wait for students to self-identify difficulties or concerns. Teachers, thus, move from didactic teaching and the dispensing information to working alongside students and facilitating learning, similar to that described by Gleadow *et al.* (1993).

## Collaboration tools

### Interactive mind maps

**Figure 3.**
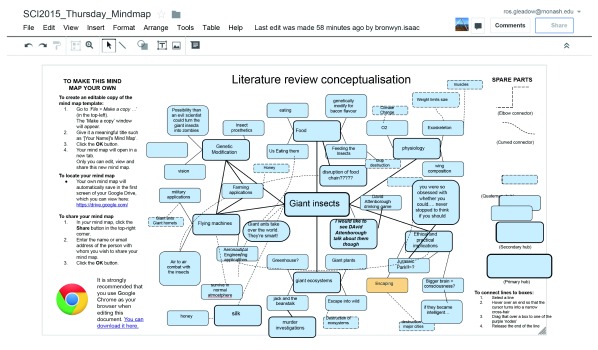
Mind mapping tool developed for students to refine and develop a research question. Here students have worked on a practice, plus topic about insects. Student can download a clean copy for personal use. A video showing how the ideas develop and the map builds is given in the supplementary information.

An important breakthrough for education is in the development of new tools that when used online allow collaboration across time and space. Our university moved to using Google-administered email accounts for all students and staff in 2010, fundamentally changing the way we were able to work with these collaborative tools. As part of a module on conceptualisation, we introduced brainstorming strategies, and as an example of this, mind-mapping. To facilitate discussion and exploration and to highlight the power of this method we built a tool to allow collaborative brainstorming both in real-time in classrooms and later via the unit’s LMS. The tool is a simple template built in Google Drawings, as shown in Figure 3. The workshop effectively introduces the student to the idea and benefit of mind mapping and also allows students to gain familiarity with the tool itself. Links to copies of the template are made available for the tutors to share with their class via the LMS. During the workshop the students use both electronic whiteboards and their personal laptops to access the template via their university Google account. Each student in the class then has an opportunity to collaborate in real-time simultaneously. (A video showing how this looks in practice can be viewed in the supplementary information.) This fast-paced activity has proven to be highly successful across the entire cohort with strong engagement from students and reports from tutors that the tool was easy set up and use in class. After the workshop, students are then able to access, again via the LMS, a copy of the mind-mapping template for their own use. Examination of the LMS records suggests that the majority of the students take advantage of this tool. This demonstrates the power and flexibility of adapting emerging tools in the rapidly changing digital space to enhance our students’ learning experience.

### Blogs as a tool for reflective learning

Reflective learning has always been an important part of the program for off-campus students helping the tutor to identify gaps in understanding and promote discussion within the group. This was done by using threaded discussion boards on the LMS, and while extremely valuable, the threads could become very unwieldy as people commented in posts within posts, and so on. As blogging had previously been reported to be an effective way to promote learning and understanding ( Farmer *et al.*, 2008), we explored this as an alternative to the discussion boards. Fortunately, the LMS that we use contains a blogging module that we could implement simply to both to encourage reflection on the lecture content, and to allow students to create their own knowledge platform. We now incorporate the blogging activity into the assessment of small group of students (typically 40 per semester) who are enrolled in an advanced version of the unit (by this stage we were phasing out our off-campus program). This activity requires students to make blog posts about aspects of the theoretical content of interest to them, and to comment on other students’ posts and discussions. When the blog was first implemented, the students were asked to make four posts during the semester and at least one comment.

Initially not all students could see the value of the blogs, as demonstrated by the following comments in the feedback from the 2013 cohort:

- *A better explanation as to how the blogs are to be done would be great. … I had no idea what I was doing when I wrote my first one.*
- *… blogs were kind of annoying rather than helpful*

This year, to encourage discussion, and to thereby assist the students in seeing the “point” of blogging, comments were also rewarded with marks. This led to much greater engagement with the blog, and many students seemed to not only understand the purpose of the blog, but to enjoy the lively discussions online. The students have had extensive discussions on lecture content ranging from logic to pseudoscience, to ethics in research. The students initiate these discussions themselves, commenting on posts to add information, ask questions or challenge points. The blog posts themselves, and the discussions that followed, gave many students an opportunity to identify concepts they were interested in, share their enthusiasm for a topic with an audience, develop their critical thinking and research skills in order to make meaningful comments, find points to challenge or logical responses and practice their scientific writing skills at the same time. The discussions have had an added benefit: content which would not normally be covered in the unit (through lack of time or lack of knowledge about specific examples) has been presented, by the students themselves, drawing on a wide range of sources.

Feedback continues to show that students find it hard to navigate around the blogs. The most important request made by students is for notification when a peer comments on a post they have made. This feature, common in most social media applications, suggests that these digital resident students expect their LMS experience to match that of other platforms, and also that they value comments on their posts – although whether this is out of a desire to further the discussions, or merely as trigger for reward mechanisms is not clear as there is an addictive element to the desire to endlessly check comments on Facebook ( Holmes *et al.*, 2014).

Reflective learning has been important for many years, often through writing reflective journal entries or diaries, or specific reflective essays. The difference between these activities and blogs is the openness of the latter, in terms of the posts being accessible by someone other than the student and their teacher. Whereas in an individual reflection, the focus is on the learner producing something that shows the teacher they have developed new knowledge or understanding, with blogging, there is a stronger emphasis on sharing; ideas written in a blog are meant to be seen by others, and this awareness of audience can be enough to encourage deeper learning because the final result will be on display and open to ‘public’ critique. The blogs also share some benefits with the tablet computer trial mentioned above: students from culturally and diverse linguistic backgrounds, and those who are less confident to speak up in a group, have the time and access to resources they need to understand material, and to express themselves with some degree of anonymity.

## Concluding remarks

Many different developing platforms and tools have applications in teaching over the years as technology use in education keeps pace with the rapidly changing digital world (e.g. Gleadow *et al.*, 1993). Information and communication technologies are constantly changing and in order to identify new, different or better options, horizon scanning is important, to keep up-to date with how students are interacting with the world academically and socially. We also have a responsibility to make sure that our teaching does not become locked into some early 21 ^st^ century paradigm. The manner with which we engage in education needs to match and be continuous with engagement with the rest of the digital world. To this end, we have also experimented with Wikis, Twitter and citizen science platforms, with varying degrees of success. For our purposes, the current formula of in-class electronic white boards, collaborative tools built using Google Docs, Facebook, and online blogs works mainly because the tools are readily accessible, familiar to a wide range of students, and enhance student connection.

**Figure 4.**
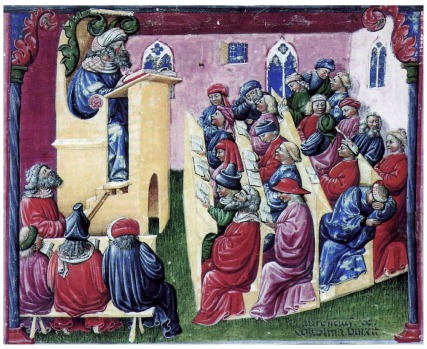
The Lecture, University of Bologna by Laurentius de Voltolina. In the 14 ^th^ Century, lectures often consisted of the lecturer reading from a book at a lecturn. Before printing, books were rare and very valuable and this made the contents more widely available. Today lectures are important in defining the area for learning and to inspire students. Note the students in the audience behave has a lot of similarities to the way to students behave in lectures today. Citation: Laurentius de Voltolina 001" by Laurentius de Voltolina - The Yorck Project: 10.000 Meisterwerke der Malerei. DVD-ROM, 2002. ISBN 3936122202. Distributed by DIRECTMEDIA Publishing GmbH. Licensed under Public Domain via Commons - https://commons.wikimedia.org/wiki/File:Laurentius_de_Voltolina_001.jpg#/media/File:Laurentius_de_Voltolina_001.jpg

Ultimately, the focus should be on what the students need to learn. We view the medium we use as secondary, and acknowledge that these tools are rapidly changing. Lectures did not end with the advent of the printing press ( Figure 4), but they did change from reading of texts to presenting knowledge with the assistance of blackboards, to overhead transparencies and then to projectors with slide slows. While digital technologies, that will themselves come and go, do enable greater control of content and information access by students, the students still need to guidance, and assistance develop the skills to use such tools safely, appropriately and confidently and we must ensure our learning objectives are not lost as we take on the challenge of keeping pace with the changing digital world.

## Supplementary materials

**Supplementary video.**

Development of a mindmap
