# Supplementary material for: New tools for a new age: An evolution or revolution in higher education?
Source: F1000Res. 2015 Dec 23;4:1502. [Version 1] doi: 10.12688/f1000research.7533.1 (PMC4712776; doi:10.12688/f1000research.7533.1)
Supplement: Peer review history

# Peer review: Referee response for version 1

**DOI:** 10.5256/f1000research.8112.r11752

**Published:** 2016-01-11

**Authors:** Mary Williams

**Affiliations:** 1 American Society of Plant Biologists, Rockville, MD, USA

I enjoyed reading this and approve it.

This is a very useful article for many instructors, particularly those who remain sceptical of the value that can be added from digital tools, and those who are interested in adopting them but unsure of how to introduce them and use them effectively. The timeline presented is interesting as it shows that, as the title suggests, big changes can take place through a series of small steps; this should be reassuring to those who hope to update their approach to teaching.

The article provides a narrative account of ten years of innovation in a large course, with explanations of what was tried, its success or not, and whether it has been continued as part of the course. I particularly appreciate the indications that tools and approaches must change as technologies and students’ experiences change – instructors must keep adopting and adapting.

A few key points:

Facebook, unlike many institutional LMSs, is optimized for mobile devices and user-friendly – even those most sceptical of social network sites should recognize that their design and technology is superior.

I appreciate that the question of “What about students/ instructors who for some reason refuse to use Facebook?” is addressed. By providing the same announcements via LMS, the students who chose to exclude themselves from the Facebook discussions are not disserviced, other than by their own choosing.

I feel that it is essential for students to develop their professional skills in a structured environment, and incorporating group work on tablet PCs and whiteboards is an excellent approach. I particularly agree with this statements, “ *Where students are given an open-ended group task, they use the technology available to them in a variety of ways that suit their learning style and expertise……Each student brings with them knowledge of different tools, so that together their power to create and innovate is far greater than them working alone*.” This is exactly the type of student-centered, problem-based learning that needs to be supported and that digital tools can support.

I’m curious, what is the topic / question they are addressing in the mindmap?

I also appreciate the distinction that is made between blogs and the reflective essays written for the instructor that are a common component of a course. It is easy to believe that the quality of the thought and presentation would be enhanced through the more open platform.

I look forward to sharing this article, as it demystifies the uses and benefits of the digital tools that ought to be a part of every instructional environment.

I have read this submission. I believe that I have an appropriate level of expertise to confirm that it is of an acceptable scientific standard.

# Peer review: Referee response for version 1

**DOI:** 10.5256/f1000research.8112.r11749

**Published:** 2016-01-08

**Authors:** Graham Scott

**Affiliations:** 1 School of Biological, Biomedical and Environmental Sciences, University of Hull, Hull, UK

This is an interesting reflection on the evolution of a programme of study through the introduction of emerging digital technologies. Although in themselves adoption of none of the technologies discussed would today be considered innovative this paper presents a useful commentary on the pros and cons of adopting each through the experiences of a teaching team who have themselves clearly been innovators in their adoption. My only concern is that in reading the paper I was left wanting more details about the technologies involved and about the evaluation of their effectiveness. I acknowledge however that this paper is an opinion piece/commentary rather than an empirical study and that the reader can access the information required if they follow the citations that are included. Should the authors feel it necessary to provide more information in a second version of the paper there are a few minor typographical errors that could be corrected (but these do not currently impact negatively upon the paper).

I have read this submission. I believe that I have an appropriate level of expertise to confirm that it is of an acceptable scientific standard.
